# Enhancing the Prefusion Conformational Stability of SARS-CoV-2 Spike Protein Through Structure-Guided Design

**DOI:** 10.3389/fimmu.2021.660198

**Published:** 2021-04-22

**Authors:** Timothy P. Riley, Hui-Ting Chou, Ruozhen Hu, Krzysztof P. Bzymek, Ana R. Correia, Alexander C. Partin, Danqing Li, Danyang Gong, Zhulun Wang, Xinchao Yu, Paolo Manzanillo, Fernando Garces

**Affiliations:** ^1^ Department of Therapeutics Discovery, Amgen Research, Amgen Inc., Thousand Oaks, CA, United States; ^2^ Department of Therapeutics Discovery, Amgen Research, Amgen Inc., South San Francisco, CA, United States; ^3^ Department of Inflammation and Oncology, Amgen Research, Amgen Inc., South San Francisco, CA, United States

**Keywords:** COVID-19, SARS-CoV-2, spike, trimer stability, prefusion

## Abstract

The worldwide pandemic caused by the severe acute respiratory syndrome coronavirus 2 (SARS-CoV-2) is unprecedented and the impact on public health and the global economy continues to be devastating. Although early therapies such as prophylactic antibodies and vaccines show great promise, there are concerns about the long-term efficacy and universal applicability of these therapies as the virus continues to mutate. Thus, protein-based immunogens that can quickly respond to viral changes remain of continued interest. The Spike protein, the main immunogen of this virus, displays a highly dynamic trimeric structure that presents a challenge for therapeutic development. Here, guided by the structure of the Spike trimer, we rationally design new Spike constructs that show a uniquely high stability profile while simultaneously remaining locked into the immunogen-desirable prefusion state. Furthermore, our approach emphasizes the relationship between the highly conserved S2 region and structurally dynamic Receptor Binding Domains (RBD) to enable vaccine development as well as the generation of antibodies able to resist viral mutation.

## Introduction

The Coronavirus Disease 2019 (COVID-19) caused by the severe acute respiratory syndrome coronavirus 2 (SARS-CoV-2) (1–3) has impacted ~100 million people worldwide and responsible for ~4 million deaths to date, according to the World Health Organization (WHO).

The sudden SARS-CoV-2 virus outbreak triggered an unprecedented response from the scientific community to halt this public health crisis. Due to urgent necessity, much of the early discovery efforts were set on similarities between this emerging virus and previous members in the betacoronavirus sub-family like SARS-CoV or MERS-CoV (4–6). An example of this is the Spike (S) glycoprotein. Like other coronavirus Spike trimers, this surface-exposed molecule is heavily decorated with N-linked glycans and mediates viral entry into the human host cell by facilitating the fusion of viral and host membranes (7). For successful membrane fusion, the SARS-CoV-2 Spike protein must undergo a considerable quaternary re-arrangement (pre- and postfusion) to enable the binding of the receptor binding domain (RBD) to the angiotensin converting enzyme 2 (ACE2) on the cell surface (5, 8, 9). The RBD is part of the S1 subunit and the quaternary arrangement of these domains comprises the apex of the S trimer. In the “closed” prefusion state, all three RBDs of the S trimer are packed tightly together and unable to bind ACE2. However, for successful viral infection of the host cell, the RBD must transition to an “up” position for engagement with the ACE2 protein (10). In context of the global COVID-19 pandemic, the dynamic nature of the RBD and Spike protein represent significant challenges for therapeutic development. For example, attempts to use the isolated RBD or an unstable Spike trimer as an immunogen may lead to the selection of non-neutralizing antibodies or ineffective vaccines (11–13).

Like with many other viruses, the prefusion state of the Spike trimer is proposed to be the most suitable target for the development of therapeutics to inhibit viral machinery and prevent infection (e.g., neutralizing antibodies, vaccines, or even small molecules) (14–16). However, the prefusion state is generally transient and very unstable, as success of the virus depends on the rapid opening of the RBD trimer apex for ACE2 binding. This is then followed by the dramatic structural rearrangement of the S2 subunit required for viral-host membrane fusion (5, 6, 9, 17, 18). Therefore, there is high interest in locking the S trimer into the prefusion conformation. For example, in the HIV-Env protein, the combination of proline insertions in the gp41 subunit and disulfide bonds linkages within interdomains successfully enabled trimer stabilization and consequent rational vaccine development (19–21). A similar approach was taken for other Spike proteins through the insertion of two prolines in the S2 subunit, which prevented the prefusion conformation of the SARS-CoV and MERS-CoV Spike trimers from transitioning to the postfusion state and infecting host cells (22, 23). However, these proline mutations did not appear to inhibit RBD motion in SARS-CoV-2 (24), suggesting further modifications may be needed to achieve conformational stability.

Here, we sought to stabilize the prefusion state of the SARS-CoV-2 Spike protein through a structure-guided approach to identify candidate disulfide bonds linking the S1 to the S2 subunit. From these in-silico designs, we generated and tested a comprehensive panel for desired assembly and activity by ACE2 binding, pseudovirus fusion, and finally electron microscopy characterization. This panel resulted in multiple prefusion stabilized designs with varying degrees of conformational stability, revealing a novel mechanism for the role of RBD motion in the transition from the prefusion to postfusion state of the SARS-CoV-2 Spike protein.

## Material and Methods

### Design Workflow for SARS-CoV-2 Spike Variants

Structural modeling of the SARS-CoV-2 trimer was performed using RosettaScripts and the Talaris2014 score function (25). Using the “fastrelax” protocol, five cycles of backbone minimization and rotamer optimization brought the PDB 6VXX template structure to a local energy minimum. After relaxation, we selected residues with a Cβ atom within 6.5Å of an S1 Cβ atom in different protomers, resulting in 56 potential disulfide candidates. For each potential disulfide, Cysteines were computationally introduced for both residues and forced into a disulfide. This was followed by 50 Monte Carlo based simulated annealing steps for the peptide backbone and surrounding residues. The final models were ranked relative to each other using the Talaris2014 score and the unweighted disulfide potential term. 9 disulfides were selected, and disulfides cross-linking distinct domains were paired into double disulfide pairs, for a total of 33 unique designs.

The SARS-CoV-2 S-PP variant served as the case construct for all designs (6). Briefly, the base construct consisting of residues 1-1208 of the SARS-CoV-2 S protein (YP_009724390.1) with prolines introduced at residues 986 and 987, “GSAS” substituted at the furin cleavage site at residues 682-685, and a C-terminal fibritin trimerization motif, HRV3C protease site, octa-histidine tag and two Strep-tags was cloned into the pTT5.2 vector. Each of the 33 designs was introduced into this construct and an identical construct without the PP mutations. Finally, 2 additional constructs were generated without the GSAS substitution site and fibritin trimerization domain as controls for a total of 70 unique constructs.

### Recombinant Protein Expression

Plasmids encoding the Spike variants were transiently expressed in suspension human embryonic kidney 293-6E cells (NRC-BRI). Briefly, cells were maintained in FreeStyle F-17 medium (Thermo Fisher) with 0.1% Kolliphor P188 (Sigma), 25 μg/ml G418 (Gibco) and 6mM L-glutamine (Invitrogen). To achieve a density of 2x10^6^ viable cells per ml for optimal transfection, cells were passaged 26 hrs in advance. For each ml of cells, 0.5 μg DNA was complexed with 1.5 μl PEImax reagent (Polysciences) in 100 μl FreeStyle F-17 medium for 10 min, and then added to cell culture. One day after transfection, cell cultures were fed with Tryptone N1 solution (Organotechnie) and glucose (Thermo Fisher) to a final concentration of 2.5 g/L and 4.5 g/L, respectively. Three days later, 3.75 mM sodium valproate (MP Bomedicals) was added to enhance protein expression. At day 6 post transfection, conditioned medium was harvested for purification.

### Purification

Clarified and filtered culture media were applied to HisTrap Excel 5 ml column (Cytiva) at 3 ml/min followed by 10 CV wash with 50 mM sodium phosphate, 300 mM NaCl, 10 mM imidazole, pH 8.0. Bound proteins were eluted with 50 mM sodium phosphate, 300 mM NaCl, 500 mM imidazole, pH 8.0. Pooled eluate was loaded onto Strep-Tactin Superflow Plus cartridge (1 ml or 5 ml at 0.5 ml/min or 3 ml/min; Qiagen). The resin was washed with 10 CV of 50 mM sodium phosphate, 300 mM NaCl, pH 8.0 and proteins were eluted in the same buffer with 2.5 mM desthiobiotin. Fractions containing protein were pooled, concentrated in 30 kDa MWCO concentrator (Pierce) to 0.1-0.4 ml and manually injected onto Superose 6 Increase (Cytiva) equilibrated in 20 mM Hepes, 150 mM NaCl, pH 7.6. Protein concentration was determined using Nanodrop 2000 or 8000 (Thermo Fisher).

### Cell Lines

HEK293T cells were purchased from ATCC (CRL-3216) and cultured in DMEM high glucose media (Invitrogen) supplemented with 1x Glutamax (Invitrogen), 1x penicillin/streptomycin (Invitrogen) and 10% FBS (Hyclone). HEK293T cells stably expressing human ACE2 (Reference Seq: NM_021804.2) was generated using an ACE2 lentiviral expression vector from Genecopeia (EX-U1285-Lv105).

### S Protein Constructs and Expression

DNA encoding human codon optimized SARS-CoV-2 S protein (Ascension: YP_009724390.1) was purchased from Genecopeia and subcloned into the PCDNA3.1(-) expression vector (Invitrogen). S protein variants were generated using the QuickChange II XL site directed mutagenesis kit per manufacturer’s instructions. For expression of S protein, 1million HEK293T cells were transfected with 2.5µg of the indicated expression vector using Lipofectamine 3000(Invitrogen) and analyzed for experiments 24 hrs post transfection.

### Flow Cytometry Analysis (FACs) of S Protein Expression and ACE2-Fc Binding

HEK293T cells transfected with S protein expression vectors were resuspended in FACs buffer (PBS containing 0.5% bovine serum albumin and 0.05% sodium azide). For flow cytometry analysis of S protein expression, cells were stained with rabbit anti SARS-Cov-2 S protein antibody (Biovision#A3000) at a concentration of 5µg/ml on ice for 1 hr. Cells were washed and stained with anti-rabbit PE secondary antibody (BioLegend) at 1:50 dilution for 20mins before analysis. For FACs analysis of ACE2 binding, cells were incubated with human ACE2-Fc (RnD systems #10544-ZN) at 0.005µg/ml. on ice for 1hr. Cells were washed and stained with anti-Human IgG labeled with Alexafluor647 (Invitrogen) at 1:50 dilution for 20mins before analysis. Flow cytometry was performed using a FACSymphony instrument (BD Biosciences) and data analyzed using FlowJo software (BD Biosciences).

### S Protein Pseudovirus Assay

For generation of S protein containing lentiviral pseudovirus, 5.5 million HEK293T cells were plated into a 10cm dish overnight. Cells were transfected with 3.05µg of S protein vector, 8 µg of GFP-luciferase reporter vector (System Biosciences BLIV713PA-1) and 6.5µg of lentiviral packaging vector PSPAX2 using Lipofectamine 3000 (Invitrogen). Media was changed 24 hours post transfection. Viral supernatants were collected and combined 48- and 72-hours post transfection. Virus was concentrated using the LentiX concentrator (Clontech) per manufacturer’s instructions. For pseudoviral infections, HEK293T cells stably expressing ACE2 were infected with virus overnight. Media was exchanged 24 hours post viral infection. 48 hours post infection, viral entry was analyzed *via* luciferase activity using the Bio-Glo luciferase assay system (Promega) and the Envision 2103 plate reader (Perkin Elmer).

### Negative Staining, 2D Class Average and 3D Reconstruction

For negative stain EM, 3 µl of protein sample at 10-15 µg/ml was adsorbed to continuous carbon grids (CF300-Cu-UL, Electron Microscopy Sciences) and subsequently stained with 2% uranyl acetate. Images were acquired on a FEI Talos F200C electron microscope operated at 200 kV, at a nominal magnification of 22,000x (corresponds to 1.84 Å/pixel) using a Gatan K3 camera. Samples were stored at 4°C up to 27 or 12 days and imaged to assess the protein stability. Image processing was carried out within the Relion 3.0 software package (26). Briefly, Laplacian-of-Gaussian algorithm was used for automatic particle picking from the CTF corrected images, the picked particles were cleaned through reference-free 2D classification to remove contaminants and bad particles. The cryo-EM structure of S-protein (EMD-21452, Walls et al., 2020) was stripped of RBD information and low-pass filtered to 60 Å resolution for use as a model for 3D classification and 3D autorefinement.

## Results

### Rational Design of a Stabilized SARS-CoV-2 Spike Trimer

**Figure 1:**
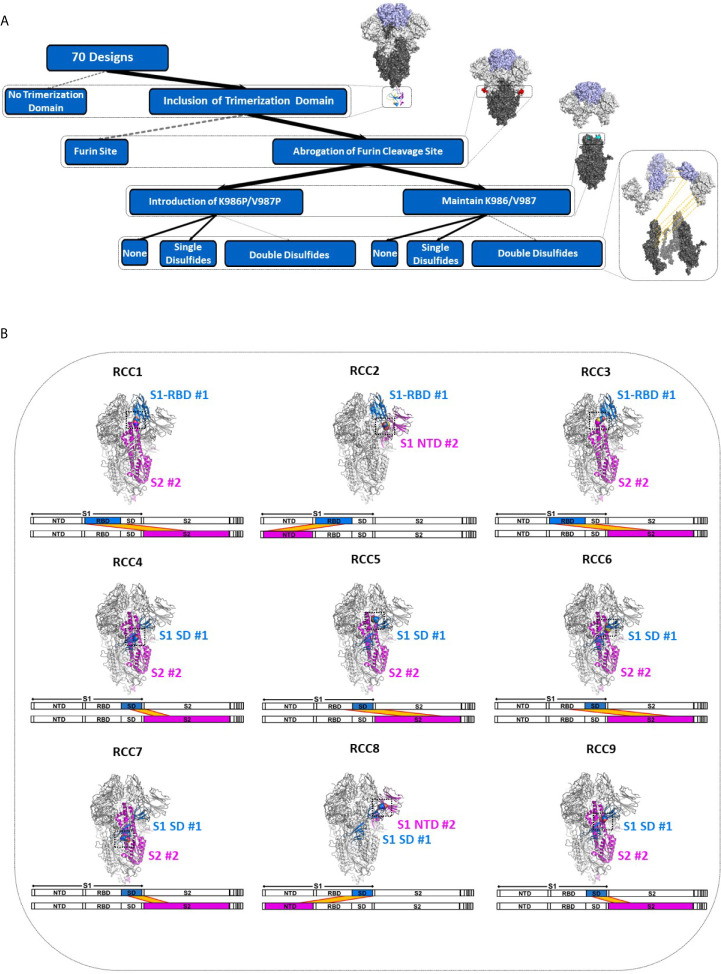
Deconstruction of the SARS-CoV-2 Spike Trimer. Flowchart representation of the experimental variables in this study consisting of (top to bottom): trimerization domain, furin site, K986P/V987P prefusion locking mutations, and rationally designed disulfides **(A)**. Structural models of each rationally designed disulfide, with sequence maps shown below each model **(B)**. In **(B)**, engineered disulfides are shown as spheres in each structural model, and the disulfide-linked regions are indicated in each sequence map by a yellow band.

In order to explore the impact of RBD motion on the Spike trimer and prefusion status, we investigated the insertion of possible covalent disulfide bonds with the express goal of cross-linking domains in the S1 subunit. Thus, we computationally screened over 900,000 residue pairs in the previously determined prefusion stabilized Cryo-EM structure (containing the K986P/V987P mutations, referenced here as Spike_PP) (5) and designed new disulfides at sites where the Cβ distance <6.5Å. The *in silico* search yielded 9 distinct disulfides cross-linking the N-terminal domain (NTD), RBD, or sub-domain (SD) of the S1 subunit to other domains within the entire Spike_PP protein (i.e., RBD-NTD, RBD-S2, SD-S2; **Figure 1**). We ranked candidate disulfides according to Rosetta Energy Units (REU) and classified each design by cross-linking strategy (**Table 1**). We further paired disulfides by cross-linking different S1 domains, resulting in 24 double disulfide designs. Additionally, each disulfide design also cross-linked the Spike protomers, potentially providing another mechanism to lock the entire trimer into the prefusion state. Of note, most designs resulted in a substantial increase in Rosetta energies relative to the parental structure (ΔREU>0; **Table 1**), indicating suboptimal mutations and thus potential liabilities for each trimer design. Although Rosetta Energy Units are arbitrary and relative, higher scores could translate to unfavorable properties such as decreased trimer yields or stability for each candidate design. The trimeric symmetry of the Spike protein further compounds this effect, as each novel disulfide is represented in triplicate for each complete Spike trimer.

**Table 1:** Properties of SARS-CoV-2 Spike Proteins.

Name	Descriptor		ΔTotalEnergy	ΔDisulfidePotential	ExpressionImpact	Trimer Formation
Spike Monomer	No trimerization domain				++++	–
Spike_Furin	Full furin Site				+++	+
		Controls				
Spike_PP	K986P/V987P; Knocked out furin site				+++	+
Spike_KV	Knocked out furin site				++	+

RCC1	V382C - R983C		4.53	-0.42	–	Undetermined
RCC2	A520C- K41C		4.76	0.37	–	Undetermined
RCC3	S383C - D985C		6.17	1.02	++	+
RCC4	D614C - T859C		7.29	0.83	+	+
RCC5	T547C- N978C	Cross-linking Designs	8.84	0.88	+	+
RCC6	A570C - V963C		8.96	3.08	+++	+
RCC7	P665C - L864C		9.07	0.82	–	Undetermined
RCC8	F562C - P225C		9.52	0.14	–	Undetermined
RCC9	P589C - F855C		9.74	1.48	–	Undetermined

Table includes the shorthand name of different Spike elements explored here, the descriptor of each design, Talaris 2014 energies, the dslf_fal3 energy term, relative expression impact (denoted as -,+ > 0 µg/L, ++ > 100 µg/L, +++ > 500 µg/L, or ++++ > lOOOµg/L), and trimer fmmation (denoted as +, -, or undetennined).

Additionally, although the previously described prefusion locked Spike protein (Spike_PP) is arguably one of the most studied SARS-CoV-2 Spike variants with multiple Cryo-EM structures (4–6, 27–29), the prefusion locking mechanism of the proline mutations at K986 and V987 is not clear. These mutations were initially introduced to stabilize the prefusion state of other coronaviruses (23) but were incompletely studied in SARS-CoV-2. Notably, structural studies indicate >50% of Spike_PP particles contain one RBD in the receptor-accessible “up” position, suggesting the functional mechanism of these mutations is not through an inhibition of ACE2 recognition (5). Furthermore, more recent data suggests the PP mutations may not confer improved stability to the SARS-CoV2 Spike protein (30). Thus, although we designed our disulfides using a Spike_PP cryo-EM structure (PDB 6VXX), we also explored the impact of each design in the Spike_KV context.

### Spike Protein Trimers Are Inherently Unstable

**Figure 2:**
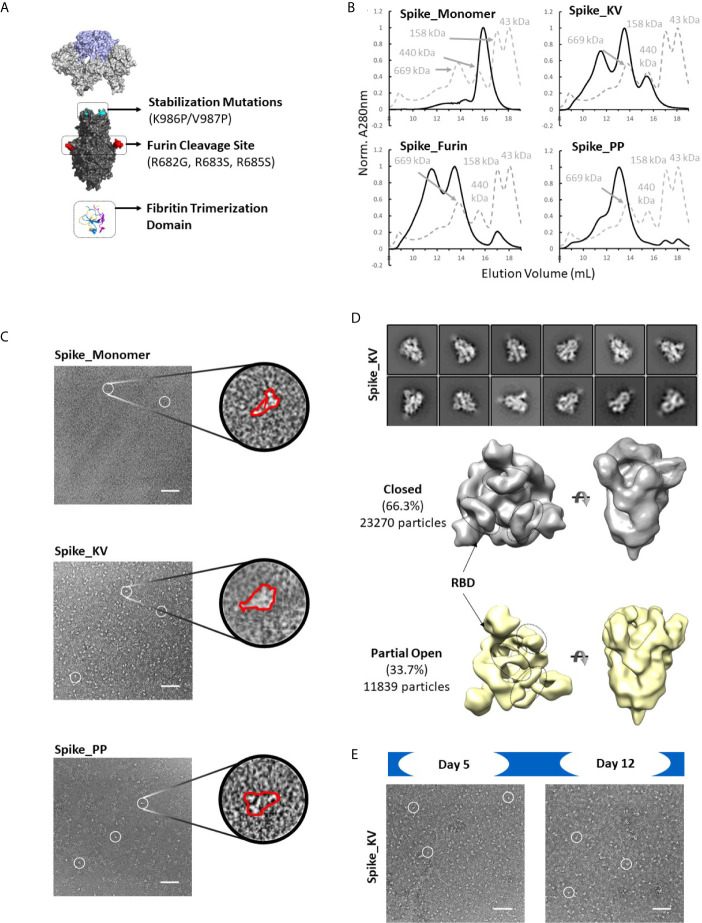
Identification of spike trimers. Expanded view of the Spike trimer, highlighting the fibritin trimerization domain, furin cleavage site and abrogation mutations, and the prefusion stabilizing mutations K986P/V987P **(A)**. The effects of stabilization mutations (Spike_PP), removal of the trimerization domain (Spike_Monomer) and re-introduction of the furin cleavage site (Spike_Furin) on the oligomerization state of the Spike protein compared to Spike_KV (Superose 6 SEC chromatograms; Standard elution traces shown in grey) **(B)**. Negative stain EM images of Spike_Monomer (top), Spike_KV (middle) and Spike_PP (bottom) showing different trimerization and stabilization properties, with zoom-in of individual particles outlined in red; **(C)**. 2D class average (above) and 3D reconstruction (below) of the Spike_KV construct reveal two distinct Spike trimer populations, closed and partial open **(D)**. Negative stain EM images of Spike_KV beyond Day 5 after purification show significant particle degradation **(E)**. Scale bars in **(C)** and **(E)** indicate 100nm, and white circles highlight individual particles.

Consistent with observations in the field (30), the SARS-CoV-2 Spike protein appears to be extremely sensitive to expression conditions. Indeed, even the control constructs without any non-native disulfides produced only a few hundred micrograms of material per liter of expression media. To select the optimal scaffold for the cross-linking disulfides, we first investigated four variants without disulfides interrogating different aspects of the previously described prefusion-locked ectodomain construct (the non-native fibritin trimerization domain, abrogation of the furin cleavage site, and the K986P/V987P mutations) (**Figure 2A**). Our findings were largely consistent with what others have found (30–32). Briefly, of the four control constructs (thus named: Spike_Monomer, Spike_Furin, Spike_KV, and Spike_PP), removal of the trimerization domain (Spike_Monomer) had the most significant positive impact on yields, producing over 3 mg/L (**Table 1**). At the other end of the spectrum were constructs containing the WT residues K986 and V987 (Spike_KV), with measured yields in the tens of micrograms. Introducing the K986P and V987P mutations (Spike_PP) tended to improve yields relative to the WT, while re-introducing the furin cleavage site (Spike_Furin) appeared to have only marginal impact (**Table 1**).

Separation by size exclusion chromatography (SEC) and negative stain EM further characterized the oligomerization state of these protein variants. In line with what others have reported (18), unpaired Spike protomers tended to migrate on the SEC column with a size comparable to a 200kDa standard, while the complete trimer migrated with a profile that appeared slightly larger than largest standard of 669kDa (**Figures 2B, C**). Using these migration trends coupled with negative-stain EM scans, we could confirm that Spike proteins without the trimerization domain (Spike_Monomer) did not form any complete trimers, existing almost entirely as unpaired protomers. In contrast, the Spike_PP, Spike_KV, and Spike_Furin all formed significant amounts of trimers with minimal free protomer, but we observed varying amounts of high molecular weight species attributed to aggregation (**Figure 2C** and **Supplementary Figure 1**). To our surprise, the Spike_KV sample had the highest percentage of high-quality trimers, allowing for a 2D class average analysis of the open and closed conformations and 3D reconstructions. From these 3D reconstructions, we determined the Spike_KV sample had a high percentage (66%) of trimers in the “closed” conformation, with the remaining percentage containing one RBD in the “up” configuration (**Figure 2D**). This high percentage of trimers in the “closed” configuration is comparable to prior reports of “prefusion-locked” trimers containing the K986P/V987P mutations (5), highlighting that the opening and closing RBDs is a dynamic and natural process in the Spike prefusion trimer. Regardless, RBD orientation did not seem to induce transition to the postfusion state (as best determined at the low resolutions), as the S2 subunit retained a similar globular structure in both classes (**Figure 2D** and **Supplementary Figure 2**). However, all samples were also quickly degrading, highlighting the known instability issues of this protein (31, 33). Indeed, we observed significant background artifacts 5 days after purification (**Figure 2E**), and even fewer suitable trimers could be collected for 3D reconstruction by day 12. Thus, the prefusion state of the Spike trimer is an inherently unstable structure, with rapid degradation in both the native (Spike_KV) and prefusion-locked (Spike_PP) form.

### Identification of Cross-Linked Spike Proteins

Based on the results above, all rationally designed mutations were incorporated with the trimerization domain and the GSAS mutations to abrogate furin cleavage. Most of the remaining 66 cross-linking designs expressed at significantly lower levels compared to any of the non-cross-linked variants described above, perhaps representative of the energetically unfavorable properties of these disulfides as predicted by Rosetta (**Table 1**). The exception to this trend was the SD-S2 cross-linking design RCC6, which yielded comparable material relative to the non-cross-linked variants. The most severely impacted were the designs containing two engineered disulfides per protomer, demonstrating the compounding negative effects of each disulfide.

**Figure 3:**
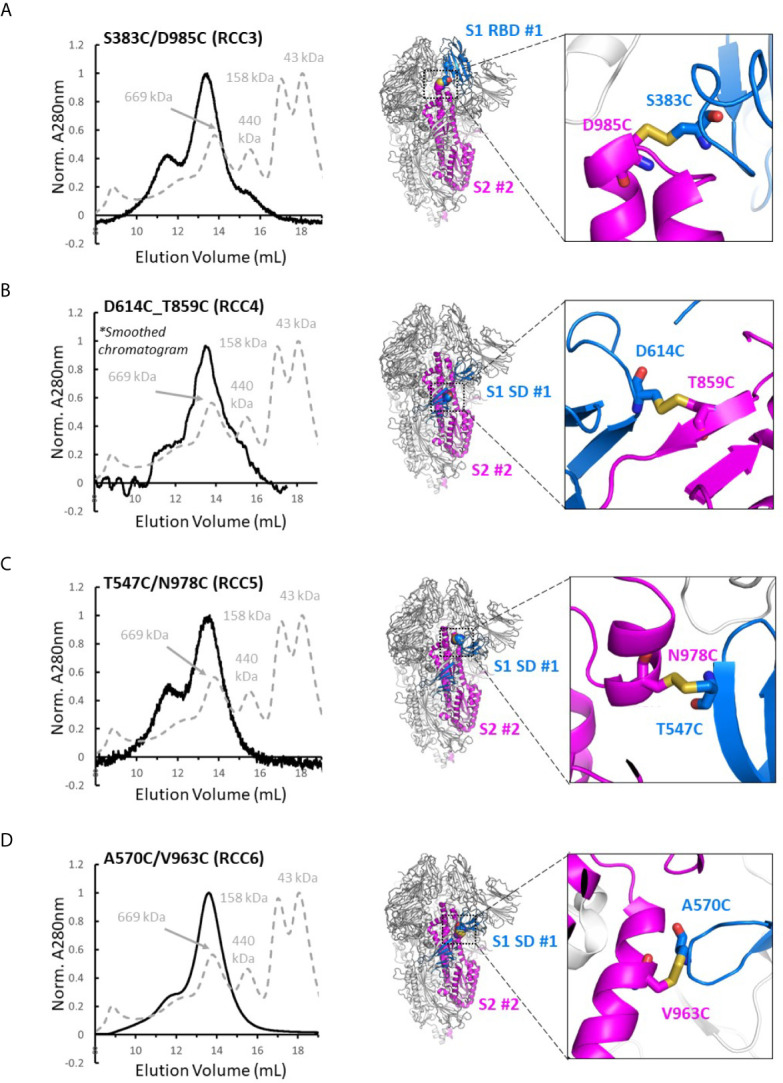
Trimer formation of rationally designed disulfides. SEC chromatograms (left) and corresponding structural models (right, with zoom-in) of each of the top four disulfide designs. RCC3 **(A)**, RCC4 **(B)**, RCC5 **(C)**, and RCC6 **(D)** each migrate on the SEC primarily as a trimer with a peak corresponding to a 669kDa MW standard (Standard elution traces shown in grey). Residues that form the engineered disulfide are shown as spheres the overall view of each model, and as sticks in each zoomed-in view.

Four disulfide designs in total (RCC3, RCC4, RCC5, and RCC6; **Figure 3**) yielded sufficient material to determine the trimeric oligomerization state by SEC. Highlighting the significance of this strategy, others have tangentially identified designs RCC3, RCC5, and RCC6 (32–34), but those characterizations were often incomplete and/or did not explore the design relationship with either the native KV residues or the stabilizing PP mutations.

Of note, none of the cross-linking designs eliminated the peak shoulder associated with higher molecular weight aggregates in non-cross-linked variants described above, further highlighting the aggregation-prone nature of the SARS-CoV-2 Spike protein (**Figure 3**). However, each design demonstrated clear trimer formation and were thus designated top cross-linking candidates for future studies.

### Cross-Linked Designs Prevent Transition to the Postfusion State

**Figure 4:**
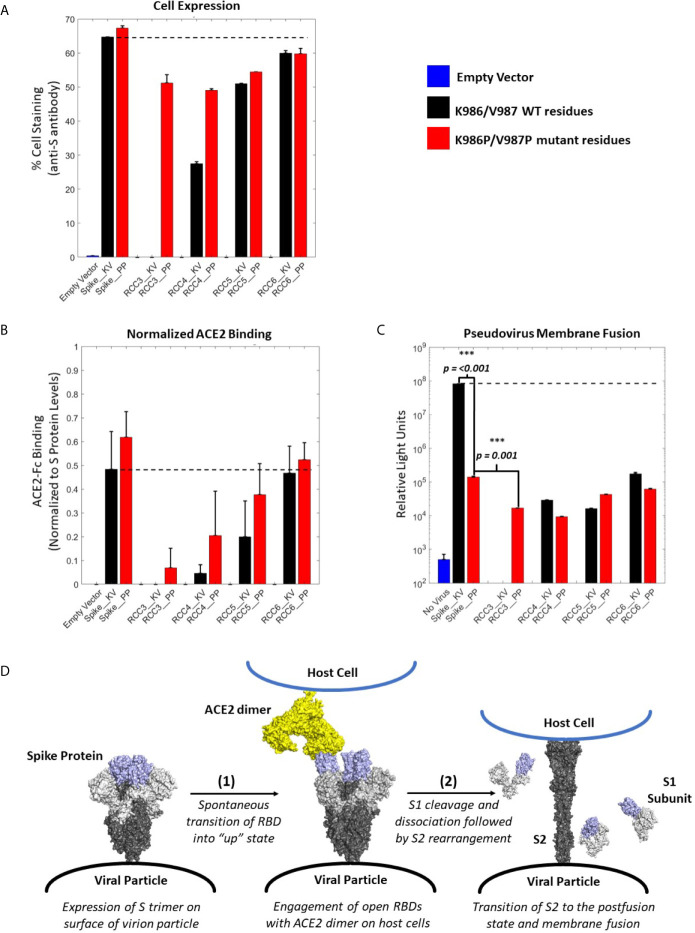
ACE2 Binding and pseudovirus inhibition. Expression of Spike trimer on cell surface of HEK293 cells as determined by staining with anti-S protein antibody **(A)**. Normalized ACE2 binding of cells expressing Spike constructs as determined by staining with ACE2-Fc antigen **(B)**. Luciferase activity of HEK293 cells infected with pseudovirus particles displaying Spike constructs with a Luciferase reporter gene. An unpaired t-test indicates the Spike_PP activity is significantly inhibited compared to Spike_KV. Additionally, p-values indicate a significant difference between Spike_PP and RCC3 as well **(C)**. Model of Spike trimer postfusion transition highlight two possible stages [(1) and (2)] for postfusion inhibition (modeled from PDB 6VSB and 6M3W) (6, 36) **(D)**. All experiments performed in triplicate with error bars denoting the standard deviation. *Note: RCC3_KV design failed cloning assembly.* ***p<=0.001.

After we identified which designs could form recombinant trimers with the trimerization domain, we explored the impact of these designs on ACE2 binding and pseudovirus membrane fusion (35). All top designs described above (RCC3, RCC4, RCC5, and RCC6) were re-cloned with the full-length transmembrane domain and expressed on the surface of HEK293 cells with or without the K986P/V987P mutations (with the exception of the RCC3_KV variant, which failed vector assembly). Staining with anti-S antibodies indicated that the various Spike proteins mimicked expression levels of recombinant proteins in culture, where all the designs except for RCC6 expressed at lower levels compared to the non-cross-linked controls (Spike_KV and Spike_PP, **Figure 4A**).

Considering the proximity of the K986P/V987P mutations with the RBDs, we considered if these mutations themselves may be shifting equilibrium to the “RBD-down” state (and thus preventing ACE2 from binding). However, cell-surface binding experiments with ACE2 demonstrated that this was not the case (**Figure 4B**). When normalized to S protein expression levels, trimers containing the K986P/V987P mutations bound a larger fraction of ACE2 compared to those without these mutations. These data further suggest that the K986P/V987P mutations do not prevent the Spike protein from engaging with ACE2 in accordance with other studies (22).

In addition to the influence of the K986P/V987P mutations, different cross-linking designs had a clear impact on the ability of the Spike protein to engage ACE2. Designs RCC3 and RCC4 had a dramatic reduction in ACE2 binding ability, suggesting these designs were successful in locking the RBD in the “down” configuration. In contrast, RCC6 demonstrated almost no reduction in ACE2 binding, perhaps indicating that this cross-linking design does not influence RBD orientation. Regardless, all cross-linking designs also reduced pseudovirus membrane fusion to statistically significant levels relative to the Spike_KV control, on par with the inhibition observed with the Spike_PP control (**Figure 4C**). Thus, we can conclude each of our cross-linking disulfide designs, regardless of ACE2 engagement, limit the ability of the S2 from undergoing the conformational shift necessary to enter the postfusion state (**Figure 4D**). However, we also observed that designs with the greatest ACE2 binding inhibition further reduced pseudovirus fusion levels (RCC3, RCC4, RCC5), while designs with minimal ACE2 blocking ability (RCC6) behaved comparably to the prefusion locked Spike_PP control. Therefore, in the proposed 2-step model for Spike protein transition states (**Figure 4D**), all designs described here (including the Spike_PP control), inhibit the dramatic structural rearrangement seen in step 2, while inhibiting ACE2 recognition in step 1 further reduces the capacity of the Spike trimer to fuse with a target cell membrane.

### Cross-Linking Has Differential Impact on RBD Motion

With the observed differences in ACE2 binding and pseudovirus behavior, we selected two disulfide designs to investigate the structural mechanisms of this phenomenon. RCC3 and RCC6 both expressed well as soluble proteins, yet they each had very different ACE2 binding profiles. Thus, these two designs were ideal candidates for a structural investigation.

**Figure 5:**
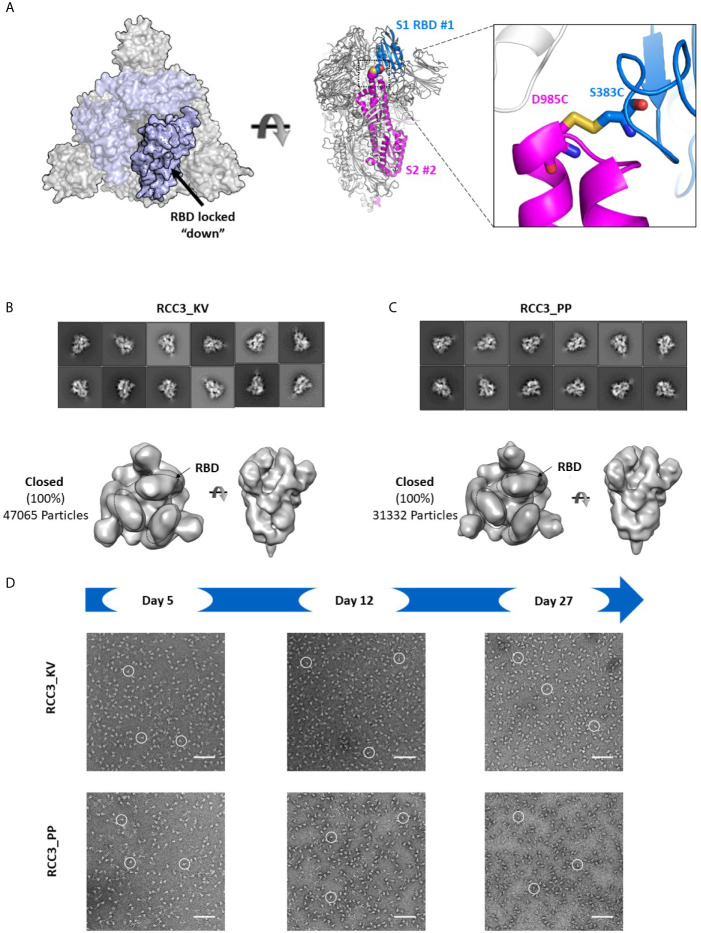
RCC3 locks All RBDs into a singular orientation. Schematic representation of RCC3 disulfide mutation expected RBD behavior (modeled from PDB 6VXX), viewed from the “top” (left) and “side” (center) and zoomed in to show the engineered disulfide (right) **(A)**. 2D class averages (top) and 3D reconstruction (bottom) of negative stain EM images of the RCC3_KV **(B)** and RCC3_PP **(C)** designs demonstrate all particles contained all three RBDs in the “closed” configuration without disrupting the S2 subunit. Spike particles were observed by negative stain EM images to be stable without significant degradation up to 27 days **(D)**. Scale bars in **(D)** indicate 100nm and white circles highlight individual particles.

Of the two disulfide designs, the design of RCC3 has been well-characterized by other groups (in context of the PP mutations) (32, 34, 37) and is designed to explicitly cross-link the RBDs to the S2 domain and lock the trimer in the prefusion “down” conformation (**Figure 5A**). Consistent with this hypothesis, we observed almost no ACE2 binding. Indeed, prior EM data (both high resolution cryo-EM and negative-stain reconstructions) of the RCC3 design in context of the PP mutations revealed nearly 100% of trimers locked the RBDs in the “down” conformation and unavailable to engage ACE2 (32). Our analysis independently confirms this behavior and extends the application of this design to the Spike KV variant without the stabilizing PP mutations (**Figures 5B, C**). Analysis of the larger complex also suggested these mutations do not disrupt the prefusion S2 structure, although minor structural changes are not detectable at these low resolutions. Thus, the cross-linking design RCC3 achieves both goals of cross-linking the Spike protomers and preventing the dynamic opening and closing of the RBD subunits.

**Figure 6:**
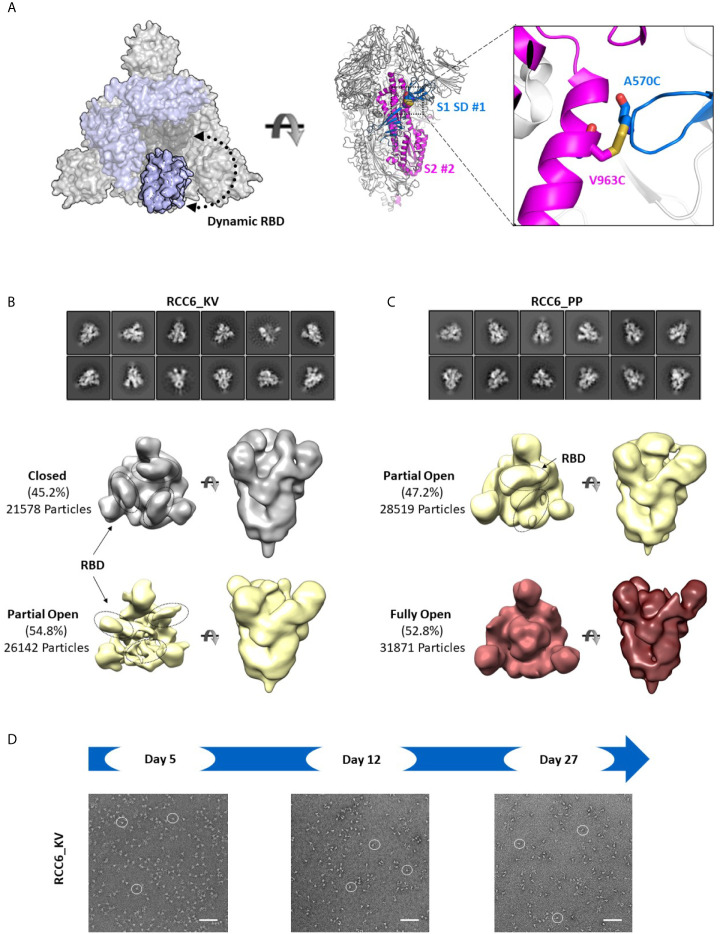
RCC6 stabilizes the prefusion state independent of RBD orientation. Schematic representation of RCC6 disulfide mutation and expected RBD behavior (modeled from PDB 6VXX and 6VSB) viewed from the “top” (left) and “side” (center) and zoomed in to show the engineered disulfide (right) **(A)**. 2D class averages (top) and 3D reconstruction (bottom) of negative stain EM images of the RCC6_KV designs shows ~45% of the particles maintain all RBDs in the “closed” configuration, while ~55% of particles are partially “open” **(B)**. In contrast, the RCC6_PP design resulted in 47% of particles in the partially “open” state and 53% in a unique “full open” state with no RBDs visible **(C)**. Both RCC6_KV and RCC6_PP had no apparent effect on S2 organization. Similar to RCC3, particles containing the RCC6 design could be identified by negative stain EM images up to 27 days with no apparent degradation **(D)**. Scale bars in **(D)** indicate 100nm and white circles highlight individual particles.

In contrast, the design of RCC6 has only been studied only at a cursory level (33), and is designed to cross-link the SD to the S2 domain with no predicted impacts on RBD motion (**Figure 6A**). In our 3D reconstructions, RCC6_KV slightly decreased the percentage of completely closed trimers when compared to the non-cross-linked Spike_KV control (45% vs. 66%, **Figure 6B**). We attributed this decrease to the cross-linked nature of the trimer, allowing the RBDs to transition to the ‘up’ conformation without completely destabilizing the quaternary structure of the trimer. Additionally, the improved trimer stability conferred by this design allowed for greater evaluation of the K986P/V987P mutations and their effect on the RBD open and closed states. Unlike with the other Spike proteins where EM identified a partial-open conformation in addition to the fully closed conformation, here with the RCC6_PP design we observed no trimers with all three RBDs in the closed conformation. Indeed, although 47% of the trimer particles had only 1 RBD “up,” the RBDs in the remaining trimers were too flexible for observation (**Figure 6C**). This increase in dynamic behavior and greater accessibility of the RBD “open” state further supports our earlier observation where the introduction of the K986P/V987P mutations in fact *increase* ACE2 binding, independent of cross-linking design (although these mutations still limit transition to the postfusion state, **Figures 4B, C**). However, as noted with the Spike_KV control, RBD orientation did not appear to affect the prefusion state as we could clearly define the S2 subunit in both orientations.

Finally, to analyze the impact of the disulfide had formed in both RCC3 and RCC6, we evaluated protein stability and protomer dissociation over time *via* negative stain EM. Compared to the non-cross-linked Spike_KV sample described above (**Figure 2E**), cross-linked samples were stable up to 27 days at 4°C with minimal dissociation or degradation (4-5 fold improvement over Spike_KV control; **Figures 5D**, **6D**), consistent with the behavior of other reported cross-linking designs (32, 33). Thus, in addition to locking the Spike trimer into the prefusion state, the designs RCC3 and RCC6 stabilize the entire prefusion trimer as well.

## Discussion

The SARS-CoV-2 virus is still relatively new and represents an urgent need to develop new therapeutic tools. Prefusion stabilization through the insertion of two proline mutations was shown to be successful in preventing postfusion transition in the SARS-CoV and MERS-CoV Spike proteins (22), and a similar SARS-CoV-2 Spike design serves as the antigen for the recent COVID-19 vaccines (which have shown up to 95% efficacy in clinical trials) (38, 39). However, due to the limited stability and conformational heterogeneity of the SARS-CoV-2 Spike trimer observed here and elsewhere (34), further improvements are needed to expand use for other applications (such as a reagent for eliciting neutralizing antibodies) (3, 4, 29, 40–42).

Here we sought a two-pronged strategy to address this limitation in the field. Through the use of rationally designed disulfides cross-linking the S2 subunit with different regions of the S1 subunit, we demonstrate the ability to simultaneously stabilize the prefusion state of the SARS-CoV-2 Spike protein with the ability to control the conformational dynamics of the RBD. Using a complete investigation including ACE2 binding analysis, pseudovirus activity, and 3D reconstructions of EM images, we are able to compare and contrast the functional behavior of different stabilized Spike constructs with the structural underpinnings of RBD dynamics. In addition to the RCC3 disulfide designed by us and others which locks the RBD into the “down” position (and thus inaccessible to ACE2) (32, 34, 37), here we present that this mutation can also be applied to the Spike_KV background with similar behavior. However, although our findings demonstrated the transferability of the RCC3 design with no clear differences in the Spike_PP and Spike_KV backgrounds, introducing the PP mutations with other disulfide designs had noticeable effects on protein behavior (with the PP mutations generally increasing RBD motion when paired with an S2 stabilizing disulfide). As a result of this, we identified designs that also stabilize the prefusion state yet also have differential impacts on the RBD dynamics (with some even *increasing* the population of trimers in the RBD “open” state, as with RCC6). As these disulfides had no obvious impact on the S2 subunit of the Spike trimers, this strategy represents a reliable method to explore the therapeutic impact of different conformational states.

Although mRNA vaccines are showing encouraging signs of success, little is known about the durability of the immune response. Therefore, significant opportunities still exist for protein vaccines and therapeutic antibodies in the future of this pandemic. We hypothesize that these stabilized trimers will be of particular use when isolating and classifying neutralizing antibodies, as the conformational state of the RBD will likely differentiate neutralizing antibodies. For example, RBD “closed” trimers may elicit antibodies to the more conserved S2 subunit (**Supplementary Figure 3**) (43) by “masking” immunodominant elements of the RBD (34), while an “open” trimer presents these immunodominant regions in context of a more relevant quaternary structure (unlike the free RBD) (44). Such tools represent an opportunity to target specific regions and epitopes of the Spike protein, as flexible regions and glycans may ‘shield’ certain regions in the native trimer (45). Additionally, antibodies selected against the ‘closed’ conformation of the Spike protein may also be protective against mutational escape, as many antibody-resistant SARS-CoV-2 mutations are localized to the RBD (46, 47). Furthermore, this strategy is broadly applicable, and can likely be applied to past and future coronaviruses with similar effect.

## Data Availability Statement

The raw data supporting the conclusions of this article will be made available by the authors, without undue reservation.

## Author Contributions

Project design by TR and FG, protein design by TR and FG, protein cloning by TR and DG, protein expression by DL, protein purification by KB and AC. Gel filtration analysis by AP. SDS-PAGE analysis by DL and AC. Negative staining work and analysis by H-TC and XY. Manuscript written by TR and FG. All authors contributed to the article and approved the submitted version.

## Conflict of Interest

Authors TPR, H-TC, RH, KPB, ARC, ACP, DL, DG, ZW, XY, PM, and FG were employed by Amgen Inc.

## Supplementary Material

Supplementary Figure 1 Mapping the SEC Peak of the Spike Trimer. Evaluation of SEC fractions (indicated by purple circles) by negative stain EM and reducing SDS-PAGE gels to discriminate aggregates, trimers, and free Spike protomers. White circles highlight individual particles.

Supplementary Figure 2 Low Resolution Models of the Spike Trimer Approximate High Resolution Cryo-EM Structures. Docking the full-length Cryo-EM structure (PDB: 6VSB) into the low-resolution 3D reconstruction of the Spike_KV model **(A)**. Docking of a single protomer **(B)**. Docking of the S2 subunit **(C)**. Docking illustrations were generated using PDB 6VSB.

Supplementary Figure 3 Sequence Diversity of Coronaviruses Mapped onto the SARS-CoV-2 Spike Trimer. 1661 Coronavirus sequences (from NCBI with search parameter: Coronaviridae : Coronavirus) mapped onto the SARS-CoV-2 Cryo EM structure (PDB: 6VXX). Residues are colored by % identity according to the diagram below the structure.
