# Supplementary figures and images for: Enhancing the Prefusion Conformational Stability of SARS-CoV-2 Spike Protein Through Structure-Guided Design
Source: Front Immunol. 2021 Apr 22;12:660198. doi: 10.3389/fimmu.2021.660198 (PMC8100506; doi:10.3389/fimmu.2021.660198)

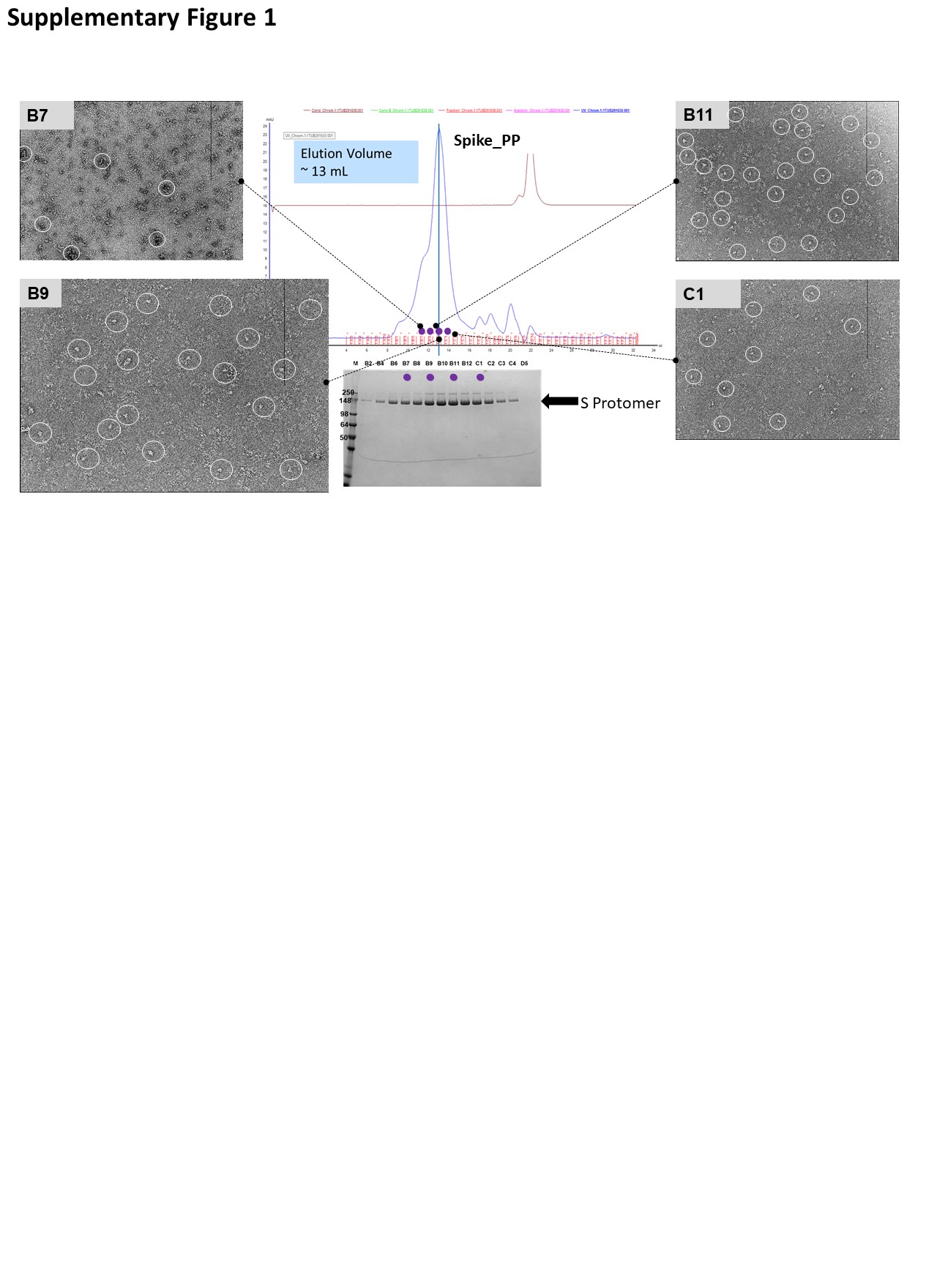

Supplement: Supplementary Figure 1 — Mapping the SEC Peak of the Spike Trimer. Evaluation of SEC fractions (indicated by purple circles) by negative stain EM and reducing SDS-PAGE gels to discriminate aggregates, trimers, and free Spike protomers. White circles highlight individual particles. [file Image_1.jpg]

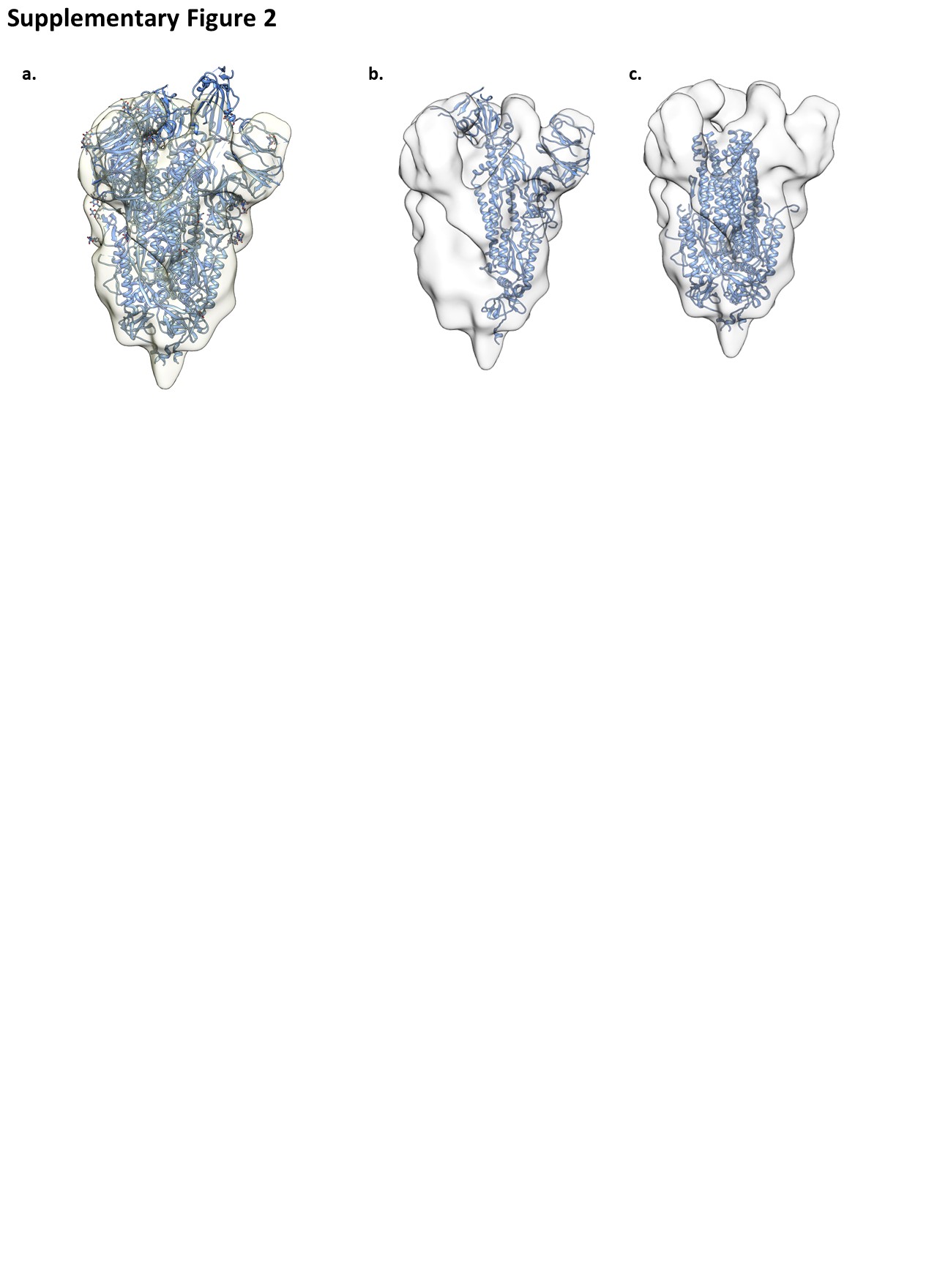

Supplement: Supplementary Figure 2 — Low Resolution Models of the Spike Trimer Approximate High Resolution Cryo-EM Structures. Docking the full-length Cryo-EM structure (PDB: 6VSB) into the low-resolution 3D reconstruction of the Spike_KV model (A). Docking of a single protomer (B). Docking of the S2 subunit (C). Docking illustrations were generated using PDB 6VSB. [file Image_2.jpg]

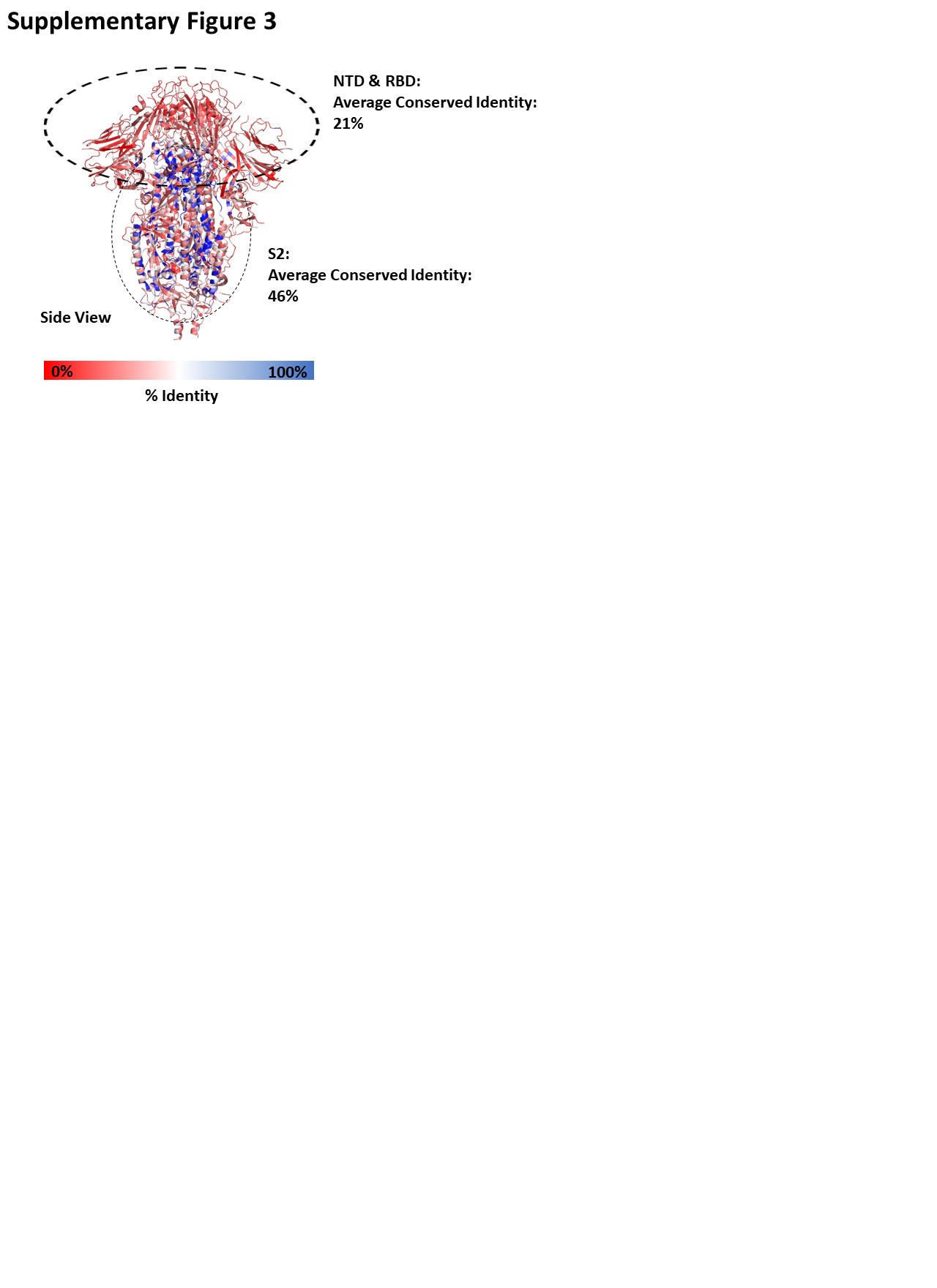

Supplement: Supplementary Figure 3 — Sequence Diversity of Coronaviruses Mapped onto the SARS-CoV-2 Spike Trimer. 1661 Coronavirus sequences (from NCBI with search parameter: Coronaviridae : Coronavirus) mapped onto the SARS-CoV-2 Cryo EM structure (PDB: 6VXX). Residues are colored by % identity according to the diagram below the structure. [file Image_3.jpg]
